# Assessing gastric cancer risk through longitudinal health check-up data: Insights from a national cohort study in South Korea

**DOI:** 10.1371/journal.pone.0312861

**Published:** 2025-04-17

**Authors:** Juwon Park, Do-young Kim, Mina Suh, Yeong-Hwa Kim, Sungho Won

**Affiliations:** 1 Trend Sensing and Risk Modeling Center, Institute of Quality of Life in Cancer, Samsung Medical Center, Seoul, Republic of Korea; 2 Department of Public Health Sciences, Seoul National University, Seoul, Republic of Korea; 3 Department of Acupuncture & Moxibustion, Jaseng Korean Medicine Hospital, Seoul, Republic of Korea; 4 National Cancer Control Institute, National Cancer Center, Goyang, Republic of Korea; 5 Department of Applied Statistics, Chung-Ang University, Seoul, Republic of Korea; 6 Institute of Health and Environment, Seoul National University, Seoul, Republic of Korea; 7 RexSoft Corps, Seoul National University Administration Building, Seoul, Republic of Korea; Gent University, BELGIUM

## Abstract

Gastric cancer (GC) is the fourth most prevalent cancer and a leading cause of cancer-related fatalities in South Korea. Although periodic screening policies are in place, the early detection and prediction of GC remain challenging. This study evaluated the risk of GC incidence by utilizing longitudinal health check-up data from the National Health Insurance Service-Health Screening Cohort spanning from 2009 to 2019. The criteria selected for this study are general health examination candidates aged 40 or older who have been eligible for health insurance since 2009. The exclusion criteria included individuals diagnosed with cancer prior to 2009 or before their examination date, as well as those who did not complete the examination questionnaire. A time-dependent Cox proportional hazards model was employed to analyze the time from health examination to the first GC diagnosis, comparing our results with previous cohort studies that evaluated the GC risk through general check-up parameters. Significant risk factors for GC incidence in both genders were age, high levels of AST and γ-GTP, low levels of ALT and hemoglobin. Among males, dyslipidemia, smoking and physical activities were also significantly associated with GC risk. Although further evidence is needed, low hemoglobin levels emerged as a promising potential risk factor for GC, ascertainable through routine general health check-ups.

## Introduction

Gastric cancer (GC) is a malignant type of upper digestive tumor that originates in the lining of the stomach [1]. In 2020, over a million new cases were diagnosed with GC, with incidence rates approximately twice as high in men compared to women [2]. Those suffering from GC experience a significantly reduced quality of life, marked by digestive disturbances, pain and poor emotional well-being due to its unfavorable prognosis. GC is the fourth leading cause of cancer-related death [3,4]. Moreover, the economic burden of curing GC in the United States (US) was estimated by the National Cancer Institute to be $2.31 billion in 2020 [5].

Accumulating evidence suggests that the causes of GC are multifaceted, including stomach infections, dysbiosis, dietary habits, obesity, smoking, alcohol consumption, and genetic factors [6]. Specifically, infection with *Helicobacter pylori* (*H. pylori*) has been primarily considered a leading cause of atrophic gastritis, accounting for over 75% of GC cases [7]. The widespread adoption of *H. pylori* eradication treatment in clinical settings has led to a gradual decrease in global GC incidence rates, a trend that is expected to continue [8]. Nevertheless, the high prevalence of GC, particularly in eastern Asia where over 60% of new cases are diagnosed, combined with the challenge of achieving a cure at advanced stages, underscores the need for developing of new strategies for early detection [9].

In South Korea, adults are entitled to national health check-ups every 2 years, while manual laborers receive these examinations annually [10]. These check-ups typically include a physician’s interview, anthropometry, basic physical examinations, and blood/urine analysis for systemic biomarkers, offering preventive care by managing potential risk factors [11]. Although upper endoscopy is the most accurate method for detecting GC, evidence supporting routine gastroscopy practice is limited, particularly in the absence of symptoms, which often do not appear until the cancer has advanced beyond its early stages [12]. Additionally, the widespread and frequent use of endoscopy across the entire population could lead to a significant societal burden [13]. Therefore, identifying high-risk groups for GC using general information becomes crucial as a preliminary step before proceeding to more invasive endoscopy procedures.

To facilitate further evidence to GC screening research, we aimed to predict GC incidence and evaluate risk factors using longitudinal health examination data from a nationwide retrospective cohort. Additionally, we conducted a review of cohort studies that have assessed GC risk using general check-up parameters. Studies analyzing GC incidence using data from large-scale general population health check-ups are limited, which hinders direct comparison of our findings with those from previous research.

## Methods

### 1. Cohort study

#### 1.1. Data source.

The National Health Insurance Service-Health Screening Cohort (NHIS-HEALS) is based on information obtained through the national health screening programs in Korea since 1995. The NHIS has provided biennial health screening (annual for manual workers) aimed at improving the health of Koreans through disease prevention and early detection [14]. The study cohort consists of health insurance subscribers and medical aid recipients as of 2002, who were in the age range of 40 to 79 years old in 2002-2003 and who received general health check-up provided by the National Health Insurance Corporation. The data comprises 514,866 individuals, randomly extracted from those who underwent health check-up, and is considered nationally representative, sampling approximately 10% of the entire Korean population. This dataset contains socioeconomic variables, health resource utilization status, disease type, clinical status and death records. Cohort participants were followed from 2002 until December 31, 2019, with no additional participants enrolled after 2002.

#### 1.2. Definition of input variables.

Information on medical examinations, blood tests, urinalysis, lifestyle check-ups such as cigarette smoking, alcohol consumption, physical activity, history of diseases, and family history of diseases was collected based on self-reported questionnaires. Smoking habits were categorized into non-smokers (individuals who had never smoked, or had smoked less than 100 cigarettes in their lifetime) and ever-smokers (individuals who had smoked in the past and who currently smoke). Alcohol consumption was classified into 3 groups: non-drinking, mild, and heavy drinking groups (defined as males consuming more than 4 drinks per week and females consuming more than 2 drinks per week). Physical activity was stratified into 3 groups based on weekly exercise frequency: non (individuals who do not work out), rare (individuals who rarely do physical activity), and active (individuals who exercise over 5 per week). To select appropriate features for building the development model, features with more than 30% missing values across all cases were removed. Additionally, subjects with missing values or outliers were eliminated from the data. Final input features are presented in Table 1.

**Table 1:** Variables based on health check-up items used in the model.

Variables
Demographic features & physical examination
Age group at the health screening date
Gender
Body mass index (BMI)
Waist circumference (WC)
Systolic blood pressure (SBP)
Diastolic blood pressure (DBP)
Blood/urine analysis
Fasting blood glucose
High-density lipoprotein cholesterol (HDL)
Low-density lipoprotein cholesterol (LDL)
Triglyceride
Hemoglobin
Creatinine
Aspartate aminotransferase (AST)
Alanine transaminase (ALT)
γ-glutamyl transpeptidase (γ-GTP)
Physician’s interview & questionnaire
History of hypertension (HTN)
History of diabetes mellitus (DM)
History of dyslipidemia
Family history of HTN
Family history of DM
Smoking habit
Alcohol consumption
Physical activity

#### 1.3. Study design.

The criteria for this study included individuals aged 40 or older from NHIS-HEALS who were eligible for health insurance and had undergone general health examinations since 2009. This year was chosen because major examination guidelines and questionnaire format in Korea were changed due to the reorganization of the health check-up system [14]. For example, variables such as triglyceride, HDL (high density lipoprotein) cholesterol, LDL (low density lipoprotein) cholesterol, creatinine, past daily smoking dose, current daily smoking dose, days of drinking per week, and the amount of drinking per day were collected only after 2009. A total of 461,046 individuals who received health examination after 2009 were selected as health insurance subscribers.

The exclusion criteria included (1) individuals diagnosed with cancer before 2009, (2) individuals diagnosed with cancer before the examination date or as a result of the examination, and (3) those who did not complete the examination questionnaire. After applying these exclusion criteria, 358,658 individuals remained eligible for the study.

**Fig 1:**
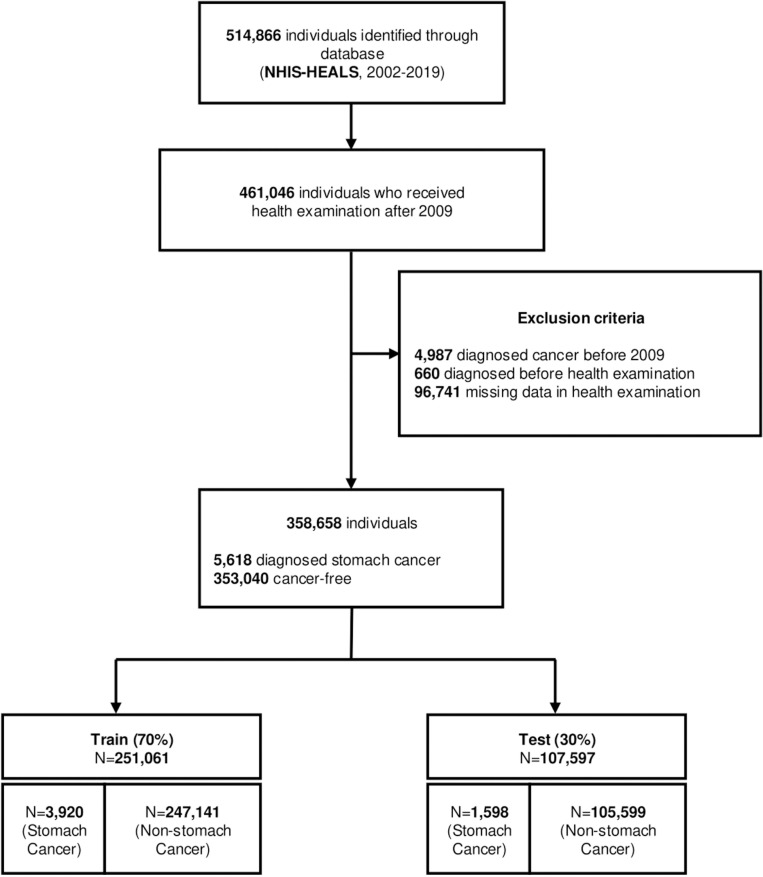
Flow chart for study population cohorts in NHIS-HEALS.

Cancer incidence was the primary outcome of this study during the follow-up period following the initial health examination date. GC, identified by the International Classification of Disease 10^th^ edition (ICD-10) code C16, was considered the first cancer detected in either the main or sub-diagnosis. The selection process for study subjects is shown in Fig 1. The study population was divided into 2 groups randomly – 70% training and 30% test dataset. The training cohort datasets were used to make a development model and fit the parameters, while the test cohort was used to assess the performance of the final models.

This study was approved by the institutional review board in Seoul National University (IRB No. E2309/002-006) and the National Health Information Data Review Committee (NHIS-2023-2-247). The requirement for informed consent was waived since the NHIS-HEALS database is anonymized administrative data.

#### 1.4. Statistical analyses.

The time-to-event was defined as the duration from the health examination date to the date of the first event diagnosis and Cox regression was performed. For individuals with multiple health check-up records, the differences between observations were accounted for using a time-dependent Cox regression model [15]. The variables selected through time-dependent Cox proportional hazard analyses, using backward selection based on AIC, are listed in Tables 2 and 3 for males and females, respectively. The general characteristics of the individuals are presented as mean ±  standard deviation for continuous variables, and as numbers (%) for categorical variables. P-values were calculated using Student’s t-test and chi-square test. A prediction model was constructed using the variables that were chosen on the training cohort datasets, and its performance was evaluated using the area under the curve (AUC) of the receiver operating characteristic (ROC) curve, where a higher AUC value indicates a better performance. Time-dependent AUCs were used to summarize predictive accuracy at specific time points, focusing on event occurrence at 1,3,5, and 7 years. All statistical analyses were performed using SAS Enterprise Guide version 7.1 (SAS Inc., Cary, NC), R version 4.3.0 (http://www.r-project.org/) software and Rex version 3.6.1.0 [16].

**Table 2:** Hazard ratios and 95% confidence intervals for the risk factors of gastric cancer in males.

Variable	Male	
HR (95% CI)	p-value
Age group		
40–49	Ref.	
50–59	**1.97 (1.23–3.16)**	**0.005**
60–69	**4.16 (2.60–6.65)**	**<0.001**
70–79	**6.18 (3.85–9.90)**	**<0.001**
≥80	**6.00 (3.64–9.90)**	**<0.001**
DBP (mmHg)	**1.00 (1.00–1.01)**	**0.040**
HDL-cholesterol (mg/dL)	**0.99 (0.99–1.00)**	**<0.001**
triglyceride (mg/dL)	**1.00 (1.00–1.00)**	**0.030**
hemoglobin (g/dL)	**0.87 (0.85–0.91)**	**<0.001**
AST (U/L)	**1.01 (1.00–1.01)**	**<0.001**
ALT (U/L)	**0.99 (0.99–1.00)**	**<0.001**
γ-GTP (U/L)	**1.00 (1.00–1.00)**	**<0.001**
History of dyslipidemia		
No	Ref.	
Yes	**0.68 (0.53–0.86)**	**0.002**
Family history of HTN		
No	Ref.	
Yes	0.89 (0.76–1.03)	0.124
Smoking habit		
Non-smoker	Ref.	
Ever-smoker	**1.31 (1.18–1.44)**	**<0.001**
Physical activity		
Non	Ref.	
Rare	**0.81 (0.71–0.93)**	**0.003**
Active	**0.82 (0.73–0.92)**	**<0.001**

DBP: diastolic blood pressure, HDL: high-density lipoprotein, AST: aspartate aminotransferase, ALT: alanine transaminase, γ-GTP: γ-glutamyl transpeptidase, HTN: hypertension, HR: hazard ratio, CI: confidence interval.

**Table 3:** Hazard ratios and 95% confidence intervals for the risk factors of gastric cancer in females.

Variable	Female	
HR (95% CI)	p-value
Age group		
40–49	Ref.	
50–59	1.28 (0.62–2.58)	0.514
60–69	**2.81 (1.39–5.70)**	**0.004**
70–79	**3.56 (1.75–7.23)**	**<0.001**
≥80	**4.56 (2.18–9.55)**	**<0.001**
BMI (kg/*m*^*2*^)	0.97 (0.93–1.00)	0.092
WC (cm)	1.01 (0.99–1.03)	0.092
HDL-cholesterol (mg/dL)	0.99 (0.99–1.00)	0.074
Hemoglobin (g/dL)	**0.82 (0.77–0.87)**	**<0.001**
AST (U/L)	**1.01 (1.00–1.01)**	**0.004**
ALT (U/L)	**0.99 (0.98–0.99)**	**0.037**
γ-GTP (U/L)	**1.00 (1.00–1.00)**	**0.035**
Family history of HTN		
No	Ref.	
Yes	**0.73 (0.58–0.93)**	**0.009**
Smoking habit		
Non-smoker	Ref.	
Ever-smoker	1.51 (0.98–2.34)	0.062

BMI: body mass index, WC: waist circumference, HDL: high-density lipoprotein, AST: aspartate aminotransferase, ALT: alanine transaminase, γ-GTP: γ-glutamyl transpeptidase, HTN: hypertension, HR: hazard ratio, CI: confidence interval.

### 2. Literature review

#### 2.1. Literature search strategy and eligibility criteria.

The literature survey was conducted across three electronic databases: PubMed, Embase, and Medline, up to January 2024. Search terms included health or health check-up, examination, gastric or stomach cancer, risk, incidence, and cohort. The search was restricted to cohort studies reporting hazard ratios (HR), and only articles in English were considered.

For literature screening, the following inclusion criteria were applied: (1) cohort studies assessing GC incidence, (2) studies involving the general population, (3) studies reporting HR with statistical analysis, and (4) independent variables comprising general check-up items such as anthropometric measures, physician’s interviews or questionnaires (e.g., medical history, smoking habit and physical activity), as well as blood, urine, and imaging tests [10]. The exclusion criteria included: (1) duplicate or incomplete articles, (2) articles not classified as cohort studies, and (3) studies not focused on evaluating the risk of GC incidence.

#### 2.2. Data extraction and assessment of statistical significance.

Data extraction encompassed the country and examination period, participant numbers, recruitment age range, variables used in the multivariate adjusted analysis, and result data from the original articles, including the statistical analyses of HR for GC incidence.

We assessed the statistical significance of the risk evaluation based on the original articles’ data presentations. The significance of the multivariate adjusted analysis was determined by non-overlapping 95% confidence intervals (CI) for the adjusted HR for GC, with a p-value below 0.05.

## Results

### 1. Cohort study

#### 1.1. The incidence of GC in the study population.

The data of 358,658 individuals were selected after excluding individuals meeting the exclusion criteria. During the study period, 5,618 cases (1.57%) of GC were identified with a median follow-up time of 8.87 years. The individuals were categorized into 2 groups: those with GC and those without GC, at any point during the follow-up period. The study population was divided into 2 groups randomly – 70% training and 30% test dataset. The training cohort comprised 251,061 individuals, among whom 3,920 GC cases were identified (2,816 male and 1,104 female). The test cohort included 1,598 GC cases (1,228 male and 470 female) out of 107,597 individuals. The baseline general characteristics of individuals in both the training and test cohorts are presented in S1 and S2 Tables.

#### 1.2. The results of risk evaluation for GC regarding general health check-up parameters.

In order to identify the possible risk factors for the incidence of the GC, time-dependent Cox proportional hazard analyses were conducted using backward selection. Tables 2 and 3 present the HR and 95% CI for each of the potential risk factor in males and females respectively.

For males, significant risk factors for GC included age, high diastolic blood pressure (DBP), low high-density lipoprotein cholesterol (HDL), high triglyceride, low hemoglobin, high aspartate aminotransferase (AST), low alanine transaminase (ALT), high γ-glutamyl transpeptidase (γ-GTP), not having history of dyslipidemia, and having smoking habits. Conversely, being active in physical activities was associated with a reduced risk of GC. The identified significant risk factors for GC in females were age, low hemoglobin, high AST, low ALT and high γ-GTP. Individuals who have family history of hypertension (HTN) showed a decreased risk of developing GC.

Using the variables selected from the Cox regression model in training cohort, the model was applied to the test cohort. S1 and S2 Figs. showed the ROC curves for cancer incidence prediction at 1,3,5, and 7 years for males and females, respectively. The AUC values for prediction years 1,3,5, and 7 were 0.667, 0.666, 0.676, and 0.673 for males, and 0.6, 0.594, 0.599, and 0.596 for females.

### 2. Literature review

#### 2.1. General characteristics of the cohort studies.

A total of 706 initial references were identified from three databases; PubMed, Embase and Medline, 18 articles were included in this review (S3 Fig). With the exception of 3 studies, the majority of the included articles were published after 2010. Half of the total studies were conducted in South Korea (5 articles) and the United States (4 articles), followed by the United Kingdom (UK) and Norway (2 articles each). The number of participants ranged from 18,244 to 6,272,367, and 10 studies (55.6%) recruited only middle-aged and older participants (≥40 years) (Table 4).

**Table 4:** Summary of the cohort studies with multivariate adjusted analysis.

Author, year	Country (examination period)	N. of participants (range of age)	Adjustments^A^	Category of independent variables	Results with statistical significance^B^ (more intense = higher risk)
Lim et al., 2022 [17]	Korea (2008-2012)	2,757,017 (N/A)	age, sex, income, smoking, drinking, exercise, BMI, DM, HTN, dyslipidemia	anthropometry	low BMI persistence of obesity
Tran et al., 2022 [18]	Korea (2002-2014)	41,837 (≥16)	age, education, family history, smoking, drinking, exercise, income, BMI	blood test	postmenopausal women: fasting glucose
Liu et al., 2021 [19]	UK (2006-2010)	465,292 (N/A)	age, ethnicity, deprivation, family history, smoking, drinking, activity, fruit, vegetable, DM, NSAID, fat/fat-free mass, height	body composition, anthropometry	whole body fat free mass men: BMI, WC
Madani et al., 2021 [20]	Iran (N/A)	47,586 (40–75)	age, marriage, education, socioeconomic status, residence, smoking, drinking, vegetable, opium, DM	anthropometry	women: WHR
Choi et al., 2021 [21]	Korea (2009–2014)	6,272,367^C^ (≥40)	menarche, parity, breastfeeding, contraceptive use, hormone therapy, menopause	anthropometry	postmenopausal women: BMI, WC
Sanikini et al., 2020 [22]	UK (2006–2010)	458,713 (40–69)	age, deprivation, education, center, smoking	anthropometry, body composition, reproductive factor	–
Sanikini et al., 2020 [23]	10 European countries (1992–2000)	476,160 (25–70)	age, center, education, smoking, BMI	anthropometry, reproductive factor	CGA: female: weight, WC, WHR CGA: male: WC NCGA: female: age at first pregnancy, bilateral ovariectomy NCGA: male: height
Hirabayashi et al., 2019 [24]	Japan (1990–1993)	92,056 (40–69)	center, family history, smoking, drinking, salt	anthropometry	men: BMI
Kim et al., 2016 [25]	Korea (2004–2007)	23,218 (≥40)	age, sex, smoking, drinking, total cholesterol	blood test	distal GC: low fasting glucose
Keum et al., 2016 [26]	US (1986)	43,479 (40–75)	age, ethnicity, family history, smoking, drinking, survey cycle, history of screening, aspirin, vitamin, total calories, meat, whole grain, fruit, vegetable, endoscopy, BMI, DM	lifestyle (activity)	–
Lin et al., 2015 [27]	Norway (1994)	192,903 (≥20)	age, sex, education, family history, smoking, BMI	blood test, anthropometry, blood pressure	metabolic syndrome, WC, non-fasting glucose female: HTN
Cook et al., 2013 [28]	US (1995–1996)	303,033 (50–71)	sex, education, ethnicity, health status, smoking, drinking, fruit, vegetable, BMI	lifestyle	NCGA: physical activity (inversely)
Doherty et al., 2011 [29]	US (1995–1996)	218,854 (50–71)	age, sex, ethnicity, total energy, marriage, education, smoking, drinking, activity, meat, fruit, vegetable, height, weight, antacid, aspirin, NSAID, DM	anthropometry	CGA: BMI, WC, hip circumference
Kim et al., 2010 [30]	Korea (1996–1997)	2,248,129 (30–80)	age, sex, family history, smoking, drinking, activity, BMI	lifestyle	salt preference
Moy et al., 2010 [31]	China (1986–1989)	18,244 (45–64)	education, smoking, drinking, preserved food, fruit, vegetable, BMI	lifestyle	NCGA: smoking
Abnet et al., 2008 [32]	US (1995–1996)	566,407 (50–71)	age, sex, education, smoking, drinking, activity	anthropometry	CGA: BMI NCGA: low BMI
Sjödahl et al., 2008 [33]	Norway (1984–1986)	73,133 (≥20)	age, activity, smoking, drinking, salt, occupation, BMI	lifestyle anthropometry	physical activity (inversely)
González et al., 2003 [34]	10 European countries (1991–1998)	521,468 (25–70)	sex, drinking, education, fruit, vegetable, meat, BMI	lifestyle	smoking

^A^These items present union of the adjusted variables for multivariate adjusted analysis, including those of had been excepted when it was independent variable.

^B^Statistical significance of multivariate adjusted analysis results was determined by non-overlapping 95% confidence intervals (CI) of adjusted hazard ratios (HR) for gastric cancer, with a p-value below 0.05.

^C^This study included women only as participants.

BMI: body mass index, CGA: cardia gastric adenocarcinoma, DM: diabetes mellitus, GC: gastric cancer, HTN: hypertension, N/A: not available, NCGA: non-cardia gastric adenocarcinoma, NSAID: non-steroidal anti-inflammatory drug, WC: waist circumference, WHR: waist-hip ratio.

#### 2.2. Results of multivariate adjusted analysis.

Among the 18 studies, six categories of independent variables were utilized to assess the risk of GC. Notably, anthropometric data such as body mass index (BMI) and waist circumference (WC) were the most frequently evaluated (11 articles, 61.1%), followed by lifestyle factors (6 articles), blood tests (3 articles), and body composition and reproductive factors (2 articles each).

Of the 11 studies incorporating anthropometric measurements, nine reported statistically significant associations between GC incidence and body characteristics, including BMI [17,19,21,24,29,32], WC [19,21,23,27,29], hip circumference [29], waist-hip ratio (WHR) [20,23], weight, and height [23], in relation to at least one subtype of GC or sex. Lifestyle variables in the included studies encompassed physical activity, salt preference, drinking, and smoking. Statistical significance was observed for smoking [31,34] and salt preference [30], while drinking [31] did not show significance, and inconsistent results were noted for physical activity [26,28,33]. In the context of blood analysis, all studies evaluating glucose metabolism-related markers found significant associations with GC when levels were above [18,27] or below [25] the normal range of blood glucose. Notable attempts of recent studies, published since the 2020s, such as body composition [19,22] and reproductive factors [22,23] did not yield consistent results with statistical significance (Table 4).

## Discussion

Through this study, we confirmed previously established risk factors for GC, such as age and smoking habits, and identified the potential of basic blood markers, such as liver function tests and hemoglobin levels, as promising risk factors. Given the asymptomatic nature or vague signs of early-stage GC, screening tests have been advocated as a secondary preventive measure, alongside risk management strategies, for early detection [35]. Countries with high prevalence and incidence rate of GC, such as South Korean and Japan, have implemented screening programs since the 2010s [36], which include periodic gastric endoscopy for middle-aged individuals and screening for *H. pylori* [37,38]. However, the societal burden of widespread screening and increase in *H. pylori*-negative cases [39,40] highlight the need to improve early detection strategies for GC, emphasizing the importance of effective risk management. This study aimed to assess the efficiency of early detection and prevention of GC by evaluating risk incidence using data from general health check-ups and conducting a comprehensive review of pertinent literatures.

As expected, the incidence of GC was significantly correlated with aging and was more predominant in males (male 4,044: 1,574 female) during 10-year follow-up period (Tables 2 and 3, S1 and S2 Tables). Similar to other types of cancer, the degeneration of cells due to accumulated stresses, such as oxidative metabolites, is believed to contribute to the development of GC [41]. Occupational environments and smoking habits have shown a high incidence rate in males, which has recently decreased with advancements in industrial medicine and anti-smoking perceptions [42]. While the exact pathophysiology remains unclear, there has been suggestion of a risk-suppressing effect of female hormones on the incidence of GC [43]. According to two studies that evaluated reproductive factors as independent variables in our review, obstetrical history or hormone therapy use failed to consistently demonstrate an association with GC (Table 4). Although one study reported that a history of bilateral ovariectomy and early pregnancy increases the risk of non-cardia gastric cancer (NCGC), hormonal effects on GC incidence remain controversial [23].

Obesity and metabolic syndrome have long been recognized as significant risk factors for gastric dysplasia, often associated with an unhealthy diet and lack of exercise [44,45]. A preventive effect of physical activity was shown in male; however, our analysis of cohort data found that neither BMI nor WC demonstrated a significant association with GC development in our cohort (Tables 2 and 3). Previous cohort studies examining the risk of anthropometry have yielded inconsistent results, varying depending on factors such as gender, GC subtype, and high/low BMI (Table 4). Based on the need for multidimensional approaches, a recent pooled analysis of cohorts in Japan indicated that while there is no clear association between BMI and NCGC, there may be with cardia gastric cancer (CGC) or esophageal cancer [46]. Similarly, recent studies published since the 2020s have attempted to analyze factors such as body composition, persistent obesity, and reproductive factors but have failed to consistently replicate significant results (Table 4).

Hyperglycemia, dyslipidemia and HTN are recognized markers of metabolic syndrome [47]. Hypothesized mechanisms linking metabolic syndrome to GC include insulin resistance-related increased insulin-like growth factor-1 availability and obesity-derived chronic inflammation [48]. However, our analysis of cohort data did not find support for an association between a history of DM or HTN and blood glucose levels with GC (Tables 2 and 3). Additionally, while our review revealed statistical significance regarding glucose levels, they were inconsistently specific to postmenopausal women, low level of fasting, and non-fasting glucose, respectively (Table 4). Similar to BMI, this variability may be attributed to GC subtypes, as it has been observed that DM is not significantly associated with overall GC but is related specifically to CGC [49]. Regarding dyslipidemia, our cohort study exhibited a notable gender-specific pattern, with a significant association observed between HDL and triglycerides in males (Table 2). Although few studies have explored the association between dyslipidemia and GC, some evidence suggests a potential role for triglycerides in the differentiation of the intestinal type of GC, which is the most common subtype and is predominant in males [50].

Due to the functions of organic metabolism and interaction with the microbiome, liver health significantly impacts both eating behaviors and gastrointestinal functions, and vice versa [51]. Our study findings indicate that elevated levels of AST and γ-GTP, along with reduced levels of ALT, are significantly correlated with the risk of GC in both males and females (Tables 2 and 3). Although the underlying pathophysiology remains incompletely understood, a poorer prognosis of GC was observed in cases where the ALT/AST ratio was ≤  0.80 compared to cases where it was >  0.80 [52]. Similarly, elevated γ-GTP levels have been identified as an unfavorable prognostic factor for liver and genitourinary cancers, as well as for DM and metabolic syndrome [53]. These findings suggest the possibility of a predictive role for liver function test not only in disease progression but also in etiology.

In our cohort data, a low hemoglobin level was associated with an increased incidence of GC in both genders (Tables 2 and 3). Pernicious anemia, a rare autoimmune disease targeting gastric parietal cells, is a well-established risk factor for NCGC [54]. However, the association of other causes of anemia with GC remains unclear. Findings from a cohort study conducted in South Korea demonstrated that anemia increases the risk of cancer in the esophagus and stomach [55]. Additionally, one study indirectly supports the role of anemia by showing the preventive effect of total iron intake for GC [56]. These findings suggest that anemia could serve as a potential marker for predicting GC risk, as it is a relatively inexpensive and readily applicable measurement.

Overall, as advancements in prevention and risk management interventions progress, the dynamics and trends of GC, including histological and demographic characteristics, become evident. For instance, a seven-fold increase in the incidence of CGC over recent decades has been attributed to heightened eradication of *H. pylori* [6]. Our findings highlight that risk factors for GC incidence are diverse, spanning gender, comorbidities, serum biomarkers, and lifestyles. This diversity underscores the importance of establishing cancer prevention strategies tailored to individual-specific characteristics. Although our AUC data for the prediction of GC may not be directly applicable to disease prediction, we believe the insights gained are invaluable. They serve as a preliminary screening tool to guide endoscopic procedures and support further investigations into GC risk factors (S1 and S2 Figs).

This study has several limitations. First, the dataset lacked information on dietary habits, such as consumption of vegetables, meats, and fried foods, which are known to be related to GC risk. The questionnaire did not contain rigorous data on eating habits. Second, we did not differentiate between GC subtypes, potentially leading to ambiguous outcomes. Third, the generalizability of our predictive results to other ethnic groups remains uncertain. Collaborative efforts involving multicenter research and external validation are imperative for further investigation. Nevertheless, this study holds significance as it comprehensively analyzed health examination questionnaires spanning the entire South Korean population. To our knowledge, studies that have assessed risk factors for GC using health check-up data, especially with blood markers, from large-scale general populations are rare. The strength of our findings lies in demonstrating the potential of basic screening parameters as early indicators before the implementation of confirmatory procedures, such as biopsy or endoscopy, for GC diagnosis.

## Supporting information

S1 Fig ROC curve for males of cancer incidence prediction after 1,3,5, and 7 years. (TIF)

S2 Fig ROC curve for females of cancer incidence prediction after 1,3,5, and 7 years. (TIF)

S3 Fig Flow chart for the literature review. (TIF)

S1 Table Baseline characteristics of the training cohort. (DOCX)

S2 Table Baseline characteristics of the test cohort. (DOCX)

## References

[1] Sitarz R, Skierucha M, Mielko J, Offerhaus GJA, Maciejewski R, Polkowski WP. Gastric cancer: Epidemiology, prevention, classification, and treatment. Cancer Manag Res. 2018;10:239–48. doi: 10.2147/CMAR.S149619 29445300 PMC5808709

[2] Morgan E, Arnold M, Camargo MC, Gini A, Kunzmann AT, Matsuda T, et al. The current and future incidence and mortality of gastric cancer in 185 countries, 2020-40: A population-based modelling study. EClinicalMedicine. 2022;47:101404. doi: 10.1016/j.eclinm.2022.101404 35497064 PMC9046108

[3] Pruthi DS, Ahmad M, Gupta M, Bansal S, Nautiyal V, Saini S. Assessment of quality of life in resectable gastric cancer patients undergoing chemoradiotherapy as adjuvant treatment. South Asian J Cancer. 2018;7(1):16–20. doi: 10.4103/sajc.sajc_196_17 29600226 PMC5865087

[4] Sung H, Ferlay J, Siegel RL, Laversanne M, Soerjomataram I, Jemal A, et al. Global Cancer Statistics 2020: GLOBOCAN estimates of incidence and mortality worldwide for 36 cancers in 185 countries. CA Cancer J Clin. 2021;71(3):209–49. doi: 10.3322/caac.21660 33538338

[5] Mariotto AB, Yabroff KR, Shao Y, Feuer EJ, Brown ML. Projections of the cost of cancer care in the United States: 2010–2020. J Natl Cancer Inst. 2011;103(2):117–28. doi: 10.1093/jnci/djq495 PMC3107566 21228314

[6] Rawla P, Barsouk A. Epidemiology of gastric cancer: Global trends, risk factors and prevention. Prz Gastroenterol. 2019;14(1):26–38. doi: 10.5114/pg.2018.80001 30944675 PMC6444111

[7] de Martel C, Ferlay J, Franceschi S, Vignat J, Bray F, Forman D, et al. Global burden of cancers attributable to infections in 2008: A review and synthetic analysis. Lancet Oncol. 2012;13(6):607–15. doi: 10.1016/S1470-2045(12)70137-7 22575588

[8] Lin Y, Zheng Y, Wang H-L, Wu J. Global patterns and trends in gastric cancer incidence rates (1988-2012) and predictions to 2030. Gastroenterology. 2021;161(1):116–127.e8. doi: 10.1053/j.gastro.2021.03.023 33744306

[9] Sekiguchi M, Oda I, Matsuda T, Saito Y. Epidemiological trends and future perspectives of gastric cancer in Eastern Asia. Digestion. 2022;103(1):22–8. doi: 10.1159/000518483 34515086

[10] Shin DW, Cho J, Park JH, Cho B. National general health screening program in Korea: History, current status, and future direction. Precis Future Med. 2022;6(1):9–31. doi: 10.23838/pfm.2021.00135

[11] Si S, Moss JR, Sullivan TR, Newton SS, Stocks NP. Effectiveness of general practice-based health checks: A systematic review and meta-analysis. Br J Gen Pract. 2014;64(618):e47–53. doi: 10.3399/bjgp14X676456 24567582 PMC3876170

[12] Zhu L, Qin J, Wang J, Guo T, Wang Z, Yang J. Early gastric cancer: Current advances of endoscopic diagnosis and treatment. Gastroenterol Res Pract. 2016;2016:9638041. doi: 10.1155/2016/9638041 26884753 PMC4739216

[13] Xia R, Zeng H, Liu W, Xie L, Shen M, Li P, et al. Estimated cost-effectiveness of endoscopic screening for upper gastrointestinal tract cancer in high-risk areas in China. JAMA Netw Open. 2021;4(8):e2121403. doi: 10.1001/jamanetworkopen.2021.21403 34402889 PMC8371571

[14] Seong SC, Kim Y-Y, Park SK, Khang YH, Kim HC, Park JH, et al. Cohort profile: the national health insurance service-national health screening cohort (NHIS-HEALS) in Korea. BMJ open. 2017;7(9). 10.1136/bmjopen-2017-016640 PMC5623538 28947447

[15] Zhang Z, Reinikainen J, Adeleke KA, Pieterse ME, Groothuis-Oudshoorn CGM. Time-varying covariates and coefficients in Cox regression models. Ann Transl Med. 2018;6(7):121. doi: 10.21037/atm.2018.02.12 29955581 PMC6015946

[16] Lee B, An J, Lee S, Won S. Rex: R-linked EXcel add-in for statistical analysis of medical and bioinformatics data. Genes Genomics. 2023;45(3):295–305. doi: 10.1007/s13258-022-01361-7 36696053

[17] Lim JH, Shin CM, Han K-D, Lee SW, Jin EH, Choi YJ, et al. Association between the persistence of obesity and the risk of gastric cancer: A nationwide population-based study. Cancer Res Treat. 2022;54(1):199–207. doi: 10.4143/crt.2021.130 33940785 PMC8756136

[18] Tran TT, Lee J, Gunathilake M, Cho H, Kim J. Influence of fasting glucose level on gastric cancer incidence in a prospective cohort study. Cancer Epidemiol Biomarkers Prev. 2022;31(1):254–61. doi: 10.1158/1055-9965.EPI-21-0670 34758969

[19] Liu AR, He QS, Wu WH, Du JL, Kuo ZC, Xia B, et al. Body composition and risk of gastric cancer: A population-based prospective cohort study. Cancer Med. 2021;10(6):2164–74. doi: 10.1002/cam4.3808 33624430 PMC7957174

[20] Madani NH, Etemadi A, Nalini M, Poustchi H, Khajavi A, Mirzazade E. et al. Obesity and incident gastrointestinal cancers: overall body size or central obesity measures, which factor matters? Eur J Cancer Prev. 2021;30(3):267. 33783379 10.1097/CEJ.0000000000000657 PMC8015184

[21] Choi IY, Choi YJ, Shin DW, Han KD, Jeon KH, Jeong S-M, et al. Association between obesity and the risk of gastric cancer in premenopausal and postmenopausal women: A nationwide cohort study. J Gastroenterol Hepatol. 2021;36(10):2834–40. doi: 10.1111/jgh.15558 34033134

[22] Sanikini H, Muller DC, Chadeau-Hyam M, Murphy N, Gunter MJ, Cross AJ. Anthropometry, body fat composition and reproductive factors and risk of oesophageal and gastric cancer by subtype and subsite in the UK Biobank cohort. PLoS One. 2020;15(10):e0240413. doi: 10.1371/journal.pone.0240413 33079929 PMC7575071

[23] Sanikini H, Muller DC, Sophiea M, Rinaldi S, Agudo A, Duell EJ, et al. Anthropometric and reproductive factors and risk of esophageal and gastric cancer by subtype and subsite: Results from the European Prospective Investigation into Cancer and Nutrition (EPIC) cohort. Int J Cancer. 2020;146(4):929–42. doi: 10.1002/ijc.32386 31050823 PMC6973006

[24] Hirabayashi M, Inoue M, Sawada N, Saito E, Abe SK, Hidaka A, et al. Effect of body-mass index on the risk of gastric cancer: A population-based cohort study in A Japanese population. Cancer Epidemiol. 2019;63:101622. doi: 10.1016/j.canep.2019.101622 31654882

[25] Kim TJ, Lee H, Min YW, Min BH, Lee JH, Son HJ, et al. Diabetic biomarkers and the risk of proximal or distal gastric cancer. J Gastroenterol Hepatol. 2016;31(10):1705–10. doi: 10.1111/jgh.13329 26936514

[26] Keum N, Bao Y, Smith-Warner SA, Orav J, Wu K, Fuchs CS, et al. Association of physical activity by type and intensity with digestive system cancer risk. JAMA Oncol. 2016;2(9):1146–53. doi: 10.1001/jamaoncol.2016.0740 27196375 PMC5846204

[27] Lin Y, Ness-Jensen E, Hveem K, Lagergren J, Lu Y. Metabolic syndrome and esophageal and gastric cancer. Cancer Causes Control. 2015;26(12):1825–34. doi: 10.1007/s10552-015-0675-4 26450604

[28] Cook MB, Matthews CE, Gunja MZ, Abid Z, Freedman ND, Abnet CC. Physical activity and sedentary behavior in relation to esophageal and gastric cancers in the NIH-AARP cohort. PLoS One. 2013;8(12):e84805. doi: 10.1371/journal.pone.0084805 24367697 PMC3868613

[29] O’Doherty MG, Freedman ND, Hollenbeck AR, Schatzkin A, Abnet CC. A prospective cohort study of obesity and risk of oesophageal and gastric adenocarcinoma in the NIH–AARP Diet and Health Study. Gut. 2011;gutjnl-2011-300551. 10.1136/gutjnl-2011-300551 PMC3504700 22174193

[30] Kim J, Park S, Nam B-H. Gastric cancer and salt preference: A population-based cohort study in Korea. Am J Clin Nutr. 2010;91(5):1289–93. doi: 10.3945/ajcn.2009.28732 20219954

[31] Moy KA, Fan Y, Wang R, Gao Y-T, Yu MC, Yuan J-M. Alcohol and tobacco use in relation to gastric cancer: a prospective study of men in Shanghai, China. Cancer Epidemiol Biomarkers Prev. 2010;19(9):2287–97. 20699372 10.1158/1055-9965.EPI-10-0362 PMC2936659

[32] Abnet CC, Freedman ND, Hollenbeck AR, Fraumeni JF, Leitzmann M, Schatzkin A. A prospective study of BMI and risk of oesophageal and gastric adenocarcinoma. Eur J Cancer. 2008;44(3):465–71. doi: 10.1016/j.ejca.2007.12.009 18221867 PMC2350215

[33] Sjödahl K, Jia C, Vatten L, Nilsen T, Hveem K, Lagergren J. Body mass and physical activity and risk of gastric cancer in a population-based cohort study in Norway. Cancer Epidemiol Biomarkers Prev. 2008;17(1):135–40. doi: 10.1158/1055-9965.EPI-07-0704 18187390

[34] González CA, Pera G, Agudo A, Palli D, Krogh V, Vineis P, et al. Smoking and the risk of gastric cancer in the European Prospective Investigation Into Cancer and Nutrition (EPIC). Int J Cancer. 2003;107(4):629–34. doi: 10.1002/ijc.11426 14520702

[35] Tan YK, Fielding JW. Early diagnosis of early gastric cancer. Eur J Gastroenterol Hepatol. 2006;18(8):821–9. doi: 10.1097/00042737-200608000-00004 16825897

[36] Conti CB, Agnesi S, Scaravaglio M, Masseria P, Dinelli ME, Oldani M, et al. Early gastric cancer: Update on prevention, diagnosis and treatment. Int J Environ Res Public Health. 2023;20(3):2149. doi: 10.3390/ijerph20032149 36767516 PMC9916026

[37] Hamashima C. Group SR, Guidelines GDGfGCS. Update version of the Japanese guidelines for gastric cancer screening. Jpn J Clin Oncol. 2018;48(7):673–83. doi: 10.1093/jjco/hyy077 29889263

[38] 현아박, 수연남, 상길이, 상균김, 기남심, 상민박, et al. The Korean guideline for gastric cancer screening. J Korean Med Assoc. 2015;58(5):373–84.

[39] Nguyen TH, Mallepally N, Hammad T, Liu Y, Thrift AP, El-Serag HB, et al. Prevalence of helicobacter pylori positive non-cardia gastric adenocarcinoma is low and decreasing in a US population. Dig Dis Sci. 2020;65(8):2403–11. doi: 10.1007/s10620-019-05955-2 31728790 PMC7220821

[40] Arnold M, Park JY, Camargo MC, Lunet N, Forman D, Soerjomataram I. Is gastric cancer becoming a rare disease? A global assessment of predicted incidence trends to 2035. Gut. 2020;69(5):823–9. doi: 10.1136/gutjnl-2019-320234 32001553 PMC8520492

[41] Berben L, Floris G, Wildiers H, Hatse S. Cancer and aging: Two tightly interconnected biological processes. Cancers (Basel). 2021;13(6):1400. doi: 10.3390/cancers13061400 33808654 PMC8003441

[42] Yusefi AR, Lankarani KB, Bastani P, Radinmanesh M, Kavosi Z. Risk factors for gastric cancer: A systematic review. Asian Pac J Cancer Prev. 2018;19(3):591. 29579788 10.22034/APJCP.2018.19.3.591 PMC5980829

[43] Camargo MC, Goto Y, Zabaleta J, Morgan DR, Correa P, Rabkin CS. Sex hormones, hormonal interventions, and gastric cancer risk: A meta-analysis. Cancer Epidemiol Biomarkers Prev. 2012;21(1):20–38. doi: 10.1158/1055-9965.EPI-11-0834 22028402 PMC3315355

[44] Golbidi S, Mesdaghinia A, Laher I. Exercise in the metabolic syndrome. Oxid Med Cell Longev. 2012;2012:349710. doi: 10.1155/2012/349710 22829955 PMC3399489

[45] Kim HY. Metabolic syndrome is associated with gastric dysplasia. Eur J Gastroenterol Hepatol. 2011;23(10):871–5. doi: 10.1097/MEG.0b013e328349aa18 21811159

[46] Koyanagi YN, Matsuo K, Ito H, Wang C, Tamakoshi A, Sugawara Y, et al. Body mass index and esophageal and gastric cancer: A pooled analysis of 10 population-based cohort studies in Japan. Cancer Sci. 2023;114(7):2961–72. doi: 10.1111/cas.15805 37013939 PMC10323111

[47] Kassi E, Pervanidou P, Kaltsas G, Chrousos G. Metabolic syndrome: Definitions and controversies. BMC Med. 2011;9:48. doi: 10.1186/1741-7015-9-48 21542944 PMC3115896

[48] Pothiwala P, Jain SK, Yaturu S. Metabolic syndrome and cancer. Metab Syndr Relat Disord. 2009;7(4):279–88. doi: 10.1089/met.2008.0065 19284314 PMC3191378

[49] Dabo B, Pelucchi C, Rota M, Jain H, Bertuccio P, Bonzi R, et al. The association between diabetes and gastric cancer: Results from the stomach cancer pooling project consortium. Eur J Cancer Prev. 2022;31(3):260–9. doi: 10.1097/CEJ.0000000000000703 34183534 PMC8709871

[50] Pih GY, Gong EJ, Choi JY, Kim M-J, Ahn JY, Choe J, et al. Associations of serum lipid level with gastric cancer risk, pathology, and prognosis. Cancer Res Treat. 2021;53(2):445–56. doi: 10.4143/crt.2020.599 33253515 PMC8053878

[51] Albillos A, De Gottardi A, Rescigno M. The gut-liver axis in liver disease: Pathophysiological basis for therapy. J Hepatol. 2020;72(3):558–77. doi: 10.1016/j.jhep.2019.10.003 31622696

[52] Chen S-L, Li J-P, Li L-F, Zeng T, He X. Elevated preoperative serum Alanine Aminotransferase/Aspartate Aminotransferase (ALT/AST) ratio is associated with better prognosis in patients undergoing curative treatment for gastric adenocarcinoma. Int J Mol Sci. 2016;17(6):911. doi: 10.3390/ijms17060911 27294917 PMC4926445

[53] Takemura K, Board PG, Koga F. A systematic review of Serum γ-Glutamyltransferase as a prognostic biomarker in patients with genitourinary cancer. Antioxidants (Basel). 2021;10(4):549. doi: 10.3390/antiox10040549 33916150 PMC8066142

[54] Murphy G, Dawsey SM, Engels EA, Ricker W, Parsons R, Etemadi A, et al. Cancer risk after pernicious anemia in the US elderly population. Clin Gastroenterol Hepatol. 2015;13(13):2282–9.e1-4. doi: 10.1016/j.cgh.2015.05.040 26079040 PMC4655146

[55] Oh TK, Song I-A. Anemia may increase the overall risk of cancer: Findings from a cohort study with a 12-year follow-up period in South Korea. Cancer Epidemiol Biomarkers Prev. 2021;30(7):1440–8. doi: 10.1158/1055-9965.EPI-20-1840 33879452

[56] Narmcheshm S, Toorang F, Sasanfar B, Hadji M, Rostami S, Zendehdel K. Association between gastric cancer and the intake of different types of iron and meats. BMC Nutr. 2023;9(1):53. doi: 10.1186/s40795-023-00688-y 36945038 PMC10029161

