# Supplementary figures and images for: Assessing gastric cancer risk through longitudinal health check-up data: Insights from a national cohort study in South Korea
Source: PLoS One. 2025 Apr 17;20(4):e0312861. doi: 10.1371/journal.pone.0312861 (PMC12005563; doi:10.1371/journal.pone.0312861)

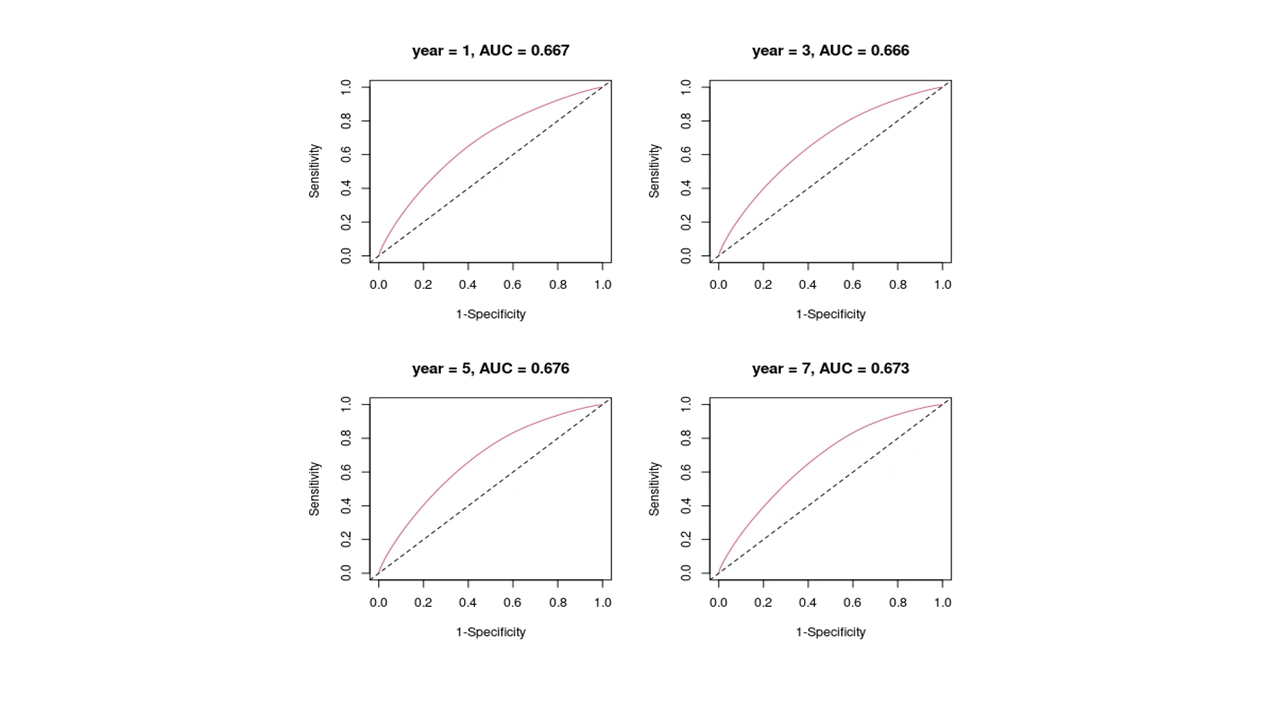

Supplement: S1 Fig — (TIF) [file pone.0312861.s001.tif]

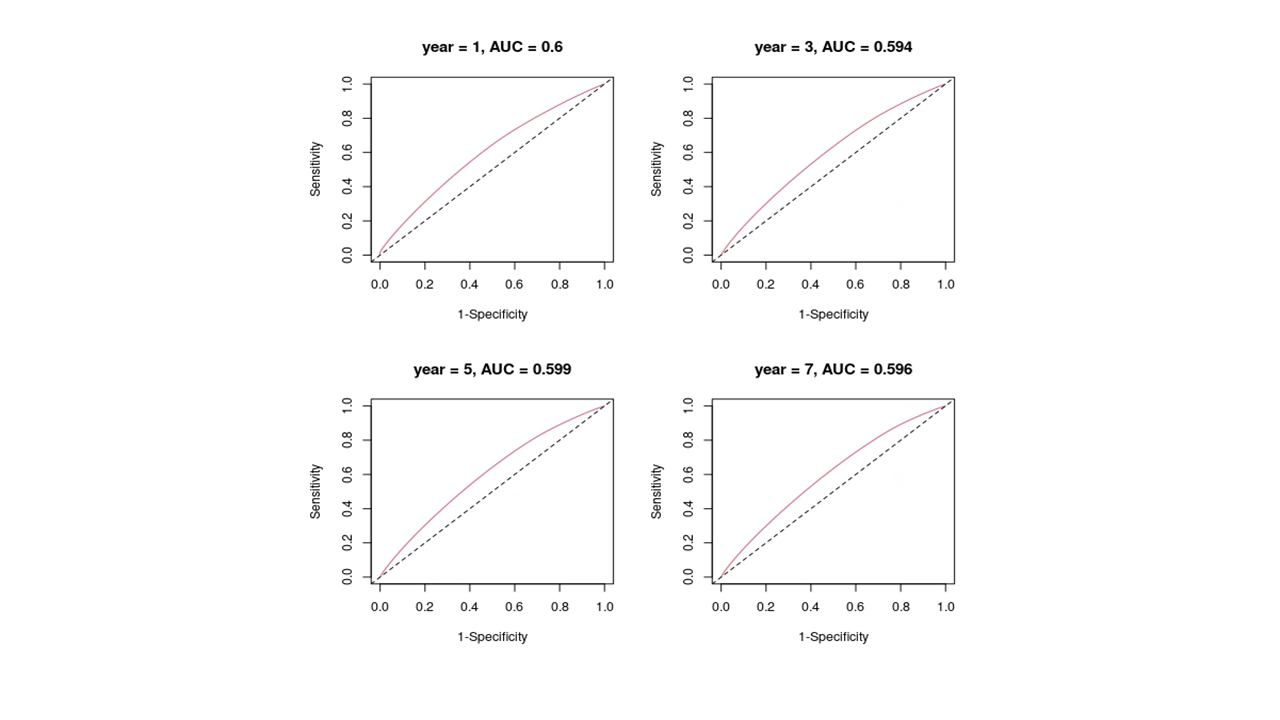

Supplement: S2 Fig — (TIF) [file pone.0312861.s002.tif]

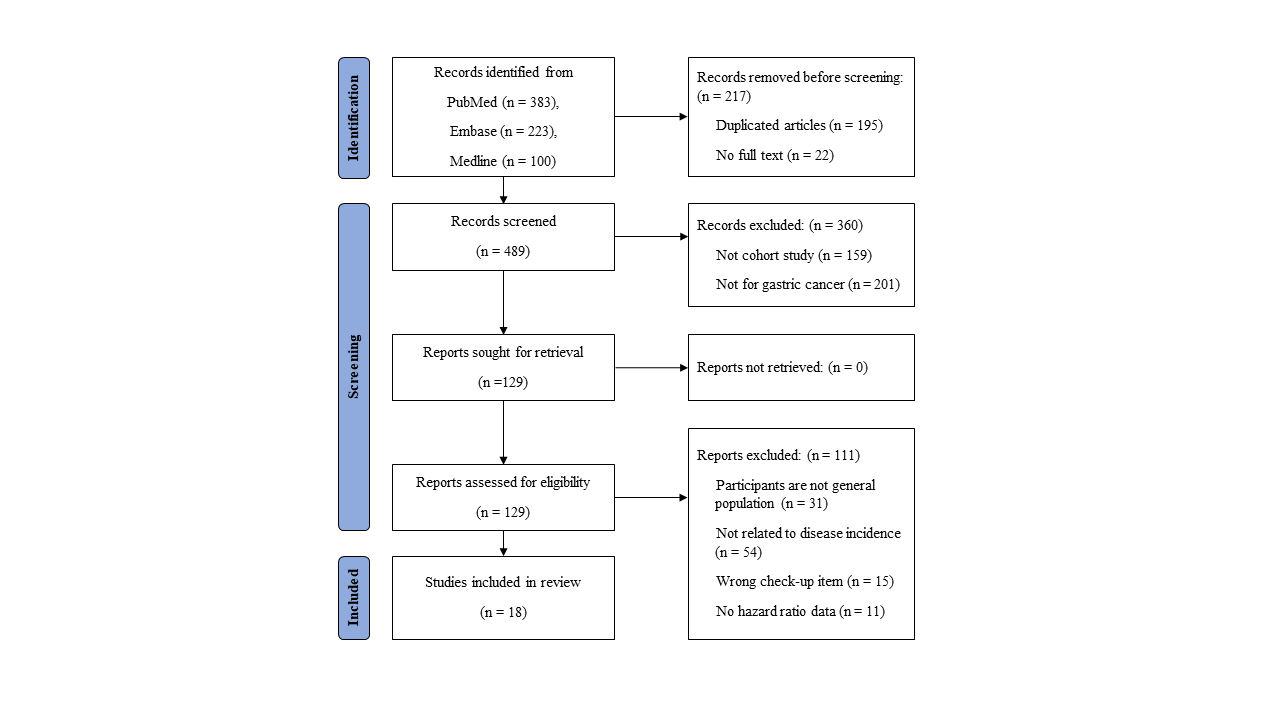

Supplement: S3 Fig — (TIF) [file pone.0312861.s003.tif]
