# Supplementary material for: Assessing gastric cancer risk through longitudinal health check-up data: Insights from a national cohort study in South Korea
Source: PLoS One. 2025 Apr 17;20(4):e0312861. doi: 10.1371/journal.pone.0312861 (PMC12005563; doi:10.1371/journal.pone.0312861)
Supplement: S1 Table — (DOCX) [file pone.0312861.s004.docx]

S1 Table. Baseline characteristics of the training cohort

| Variable | Training cohort | | | | | |
| --- | --- | --- | --- | --- | --- | --- |
|  | Male (n=133,721) | | | Female (n=117,340) | | |
|  | Gastric cancer  (n=2,816) | Non-gastric cancer  (n=130,905) | p-value | Gastric cancer  (n=1,104) | Non- gastric cancer  (n=116,236) | p-value |
| Age (years) | 63.0±8.9 | 59.7±8.7 | 0.000 | 64.9±9.2 | 60.9±9.0 | 0.000 |
| Age group |  |  | 0.000 |  |  | 0.000 |
| 40-49 | 125  (4.4%) | 11377  (8.7%) |  | 32  (2.9%) | 7356  (6.3%) |  |
| 50-59 | 949  (33.7%) | 61737  (47.2%) |  | 294  (26.6%) | 50903  (43.8%) |  |
| 60-69 | 959  (34.1%) | 35877 (27.4%) |  | 386  (35.0%) | 33640  (28.9%) |  |
| 70-79 | 684  (24.3%) | 18888  (14.4%) |  | 330  (29.9%) | 20668  (17.8%) |  |
| ≥80 | 99  (3.5%) | 3026  (2.3%) |  | 62  (5.6%) | 3669  (3.2%) |  |
| BMI (kg/$m^{2}$) | 23.9±2.9 | 24.0±2.8 | 0.017 | 24.0±3.2 | 23.9±3.1 | 0.794 |
| Waist (cm) | 84.7±7.9 | 84.4±7.5 | 0.063 | 80.1±8.2 | 79.0±8.2 | 0.000 |
| SBP (mmHg) | 78.5±9.8 | 78.1±9.6 | 0.038 | 76.3±10.1 | 75.8±9.7 | 0.146 |
| DBP (mmHg) | 127.3±14.9 | 125.8±14.4 | 0.000 | 125.2±16.3 | 123.8±15.5 | 0.003 |
| FBS (mg/dL) | 104.1±24.4 | 103.8±25.7 | 0.421 | 100.2±22.2 | 98.9±22.0 | 0.057 |
| HDL-chol (mg/dL) | 51.7±12.7 | 52.1±12.6 | 0.106 | 55.2±13.1 | 56.9±13.1 | 0.000 |
| LDL-chol (mg/dL) | 112.7±32.0 | 115.2±33.1 | 0.000 | 122.4±34.2 | 123.7±34.6 | 0.206 |
| Triglyceride (mg/dL) | 115.0±42.1 | 115.7±42.1 | 0.384 | 110.3±40.2 | 107.7±40.9 | 0.037 |
| Hemoglobin (g/dL) | 14.4±1.4 | 14.7±1.3 | 0.000 | 13.0±1.1 | 12.7±1.2 | 0.000 |
| Creatinine (mg/dL) | 1.2±1.3 | 1.2±1.3 | 0.573 | 0.9±0.8 | 0.9±0.8 | 0.941 |
| AST (U/L) | 27.6±16.5 | 27.2±16.5 | 0.207 | 25.9±20.5 | 24.9±12.6 | 0.119 |
| ALT (U/L) | 25.7±18.1 | 26.6±20.1 | 0.008 | 22.5±21.1 | 21.9±16.8 | 0.355 |
| γ-GTP (U/L) | 47.6±63.8 | 45.2±57.5 | 0.051 | 24.4±29.7 | 23.7±25.8 | 0.381 |
| History of HTN |  |  | 0.000 |  |  | 0.000 |
| Yes | 935  (33.2%) | 38584  (29.5%) |  | 413  (37.4%) | 36450  (31.4%) |  |
| No | 1881  (66.8%) | 92321  (70.5%) |  | 691  (62.6%) | 79786  (68.6%) |  |
| History of DM |  |  | 0.010 |  |  | 0.001 |
| Yes | 376  (13.4%) | 15389  (11.8%) |  | 136  (12.3%) | 10914  (9.4%) |  |
| No | 2440  (86.6%) | 115516  (88.2%) |  | 968  (87.7%) | 105322  (90.6%) |  |
| History of DYS |  |  | 0.209 |  |  | 0728 |
| Yes | 119  (4.2%) | 6221  (4.8%) |  | 84  (7.6%) | 9227  (7.9%) |  |
| No | 2697  (95.8%) | 124684  (95.2%) |  | 1020  (92.4%) | 107009  (92.1%) |  |
| Family history of HTN |  |  | 0.001 |  |  | 0.048 |
| Yes | 292  (10.4%) | 16300  (12.5%) |  | 151  (13.7%) | 18498  (15.9%) |  |
| No | 2524  (89.6%) | 114605  (87.5%) |  | 953  (86.3%) | 97738  (84.1%) |  |
| Family history of DM |  |  | 0.056 |  |  | 0.453 |
| Yes | 212  (7.5%) | 11208  (8.6%) |  | 103  (9.3%) | 11690  (10.1%) |  |
| No | 2604  (92.5%) | 119697  (91.4%) |  | 1001  (90.7%) | 104546  (89.9%) |  |
| Smoking habits |  |  | 0.013 |  |  | 0.216 |
| Non-smoker | 941  (33.4%) | 46739  (35.7%) |  | 1072  (97.1%) | 113572  (97.7%) |  |
| Ever-smoker | 1875  (66.6%) | 84166  (64.3%) |  | 32  (2.9%) | 2664  (2.3%) |  |
| Alcohol consumption |  |  | 0.001 |  |  | 0.324 |
| Non | 1190  (42.3%) | 52676  (40.2%) |  | 976  (88.4%) | 101124  (87.0%) |  |
| Mild | 722  (25.6%) | 31772  (24.3%) |  | 65  (5.9%) | 7260  (6.2%) |  |
| Heavy | 904  (32.1%) | 46457  (35.5%) |  | 63  (5.7%) | 7852  (6.8%) |  |
| Physical activities |  |  | 0.000 |  |  | 0.039 |
| Non | 664  (23.6%) | 26943  (20.6%) |  | 354  (32.1%) | 33278  (28.6%) |  |
| Rare | 638  (22.7%) | 33812  (25.8%) |  | 269  (24.4%) | 30374  (26.1%) |  |
| Active | 1514  (53.8%) | 70150  (53.6%) |  | 481  (43.6%) | 52584  (45.2%) |  |

Data given as mean ± standard deviation or number (%).

BMI: body mass index, SBP: systolic blood pressure, DBP: diastolic blood pressure, FBS: fasting blood glucose, HDL: high-density lipoprotein, LDL: low-density lipoprotein, AST: aspartate aminotransferase, ALT: alanine transaminase, γ-GTP: γ-glutamyl transpeptidase, HTN: Hypertension, DM: Diabetes Mellitus, DYS: Dyslipidemia
