# Supplementary material for: Assessing gastric cancer risk through longitudinal health check-up data: Insights from a national cohort study in South Korea
Source: PLoS One. 2025 Apr 17;20(4):e0312861. doi: 10.1371/journal.pone.0312861 (PMC12005563; doi:10.1371/journal.pone.0312861)
Supplement: S2 Table — (DOCX) [file pone.0312861.s005.docx]

S2 Table. Baseline characteristics of the test cohort

| Variable | Test cohort | | | | | |
| --- | --- | --- | --- | --- | --- | --- |
|  | Male (n=57,031) | | | Female (n=50,566) | | |
|  | Gastric cancer  (n=1,228) | Non- gastric cancer  (n=55,803) | p-value | Gastric cancer  (n=470) | Non- gastric cancer  (n=50,096) | p-value |
| Age (years) | 63.0±8.9 | 59.6±8.7 | 0.000 | 64.7±9.5 | 60.9±9.0 | 0.000 |
| Age group |  |  | 0.000 |  |  | 0.000 |
| 40-49 | 50  (4.1%) | 4906  (8.8%) |  | 23  (4.9%) | 3111  (6.2%) |  |
| 50-59 | 432  (35.2%) | 26475  (47.4%) |  | 127  (27.0%) | 21958  (43.8%) |  |
| 60-69 | 403  (32.8%) | 15225  (27.3%) |  | 143  (30.4%) | 14428  (28.8%) |  |
| 70-79 | 301  (24.5%) | 7901  (14.2%) |  | 153  (32.6%) | 9045  (18.1%) |  |
| ≥80 | 42  (3.4%) | 1296  (2.3%) |  | 24  (5.1%) | 1554  (3.1%) |  |
| BMI (kg/$m^{2}$) | 24.0±2.9 | 24.0±2.8 | 0.829 | 24.0±3.2 | 24.0±3.1 | 0.829 |
| Waist (cm) | 84.8±7.6 | 84.4±7.5 | 0.063 | 79.8±8.5 | 79.1±8.3 | 0.050 |
| SBP (mmHg) | 78.7±9.7 | 78.1±9.6 | 0.047 | 77.2±9.3 | 75.9±9.8 | 0.003 |
| DBP (mmHg) | 127.2±14.8 | 125.8±14.5 | 0.001 | 126.6±14.7 | 123.9±15.7 | 0.000 |
| FBS (mg/dL) | 104.6±24.9 | 104.0±26.2 | 0.346 | 99.1±21.4 | 98.9±21.5 | 0.847 |
| HDL-chol (mg/dL) | 51.7±12.6 | 52.0±12.6 | 0.324 | 55.0±120 | 56.9±13.2 | 0.001 |
| LDL-chol (mg/dL) | 112.5±32.3 | 115.3±33.1 | 0.003 | 123.8±34.1 | 123.8±34.8 | 0.998 |
| Triglyceride (mg/dL) | 113.7±41.7 | 115.9±42.1 | 0.067 | 113.0±40.4 | 107.6±40.8 | 0.004 |
| Hemoglobin (g/dL) | 14.4±1.5 | 14.7±1.3 | 0.000 | 12.8±1.2 | 13.0±1.1 | 0.012 |
| Creatinine (mg/dL) | 1.3±1.5 | 1.2±1.4 | 0.210 | 0.9±0.7 | 0.9±0.8 | 0.945 |
| AST (U/L) | 28.0±13.7 | 27.2±16.8 | 0.044 | 24.9±12.2 | 24.9±16.7 | 0.973 |
| ALT (U/L) | 26.6±26.1 | 26.6±20.1 | 0.964 | 21.7±19.9 | 21.9±16.6 | 0.889 |
| γ-GTP (U/L) | 47.7±60.8 | 45.0±56.2 | 0.130 | 26.3±59.1 | 23.8±26.7 | 0.358 |
| History of HTN |  |  | 0.000 |  |  | 0.001 |
| Yes | 422  (34.4%) | 16341  (29.3%) |  | 181  (38.5%) | 15645  (31.2%) |  |
| No | 806  (65.6%) | 39462  (70.7%) |  | 289  (61.5%) | 34451  (68.8%) |  |
| History of DM |  |  | 0.989 |  |  | 0.375 |
| Yes | 148  (12.1%) | 6695  (12.0%) |  | 51  (10.9%) | 4777  (9.5%) |  |
| No | 1080  (87.9%) | 49108  (88.0%) |  | 419  (89.1%) | 45319  (90.5%) |  |
| History of DYS |  |  | 0.952 |  |  | 0.803 |
| Yes | 59  (4.8%) | 2725  (4.9%) |  | 39  (8.3%) | 3947  (7.9%) |  |
| No | 1169  (95.2%) | 53078  (95.1%) |  | 431  (91.7%) | 46149  (92.1%) |  |
| Family history of HTN |  |  | 0.389 |  |  | 0.667 |
| Yes | 143  (11.6%) | 6980  (12.5%) |  | 70  (14.9%) | 7879  (15.7%) |  |
| No | 1085  (88.4%) | 48823  (87.5%) |  | 400  (85.1%) | 42217  (84.3%) |  |
| Family history of DM |  |  | 0.014 |  |  | 0.341 |
| Yes | 83  (6.8%) | 4913  (8.8%) |  | 40  (8.5%) | 4978  (9.9%) |  |
| No | 1145  (93.2%) | 50890  (91.2%) |  | 430  (91.5%) | 45118  (90.1%) |  |
| Smoking habits |  |  | 0.014 |  |  | 0.053 |
| Non-smoker | 398  (32.4%) | 20003  (35.8%) |  | 453  (96.4%) | 48998  (97.8%) |  |
| Ever-smoker | 830  (67.6%) | 35800  (64.2%) |  | 17  (3.6%) | 1098  (2.2%) |  |
| Alcohol consumption |  |  | 0.077 |  |  | 0.090 |
| Non | 530  (43.2%) | 22372  (35.8%) |  | 425  (90.4%) | 43672  (87.2%) |  |
| Mild | 289  (23.5%) | 13430  (24.1%) |  | 24  (5.1%) | 3074  (6.1%) |  |
| Heavy | 409  (33.3%) | 20001  (40.1%) |  | 21  (4.5%) | 3350  (6.7%) |  |
| Physical activities |  |  | 0.000 |  |  | 0.090 |
| Non | 318  (25.9%) | 11523  (20.6%) |  | 156  (33.2%) | 14410  (28.8%) |  |
| Rare | 274  (22.3%) | 14403 (25.8%) |  | 111  (23.6%) | 13223  (26.4%) |  |
| Active | 636 (51.8%) | 29877 (53.5%) |  | 203  (43.2%) | 22463  (44.8%) |  |

Data given as mean ± standard deviation or number (%).

BMI: body mass index, SBP: systolic blood pressure, DBP: diastolic blood pressure, FBS: fasting blood glucose, HDL: high-density lipoprotein, LDL: low-density lipoprotein, AST: aspartate aminotransferase, ALT: alanine transaminase, γ-GTP: γ-glutamyl transpeptidase, HTN: Hypertension, DM: Diabetes Mellitus, DYS: Dyslipidemia
